# Simulation-based roadmap for the integration of poly-silicon on oxide contacts into screen-printed crystalline silicon solar cells

**DOI:** 10.1038/s41598-020-79591-6

**Published:** 2021-01-13

**Authors:** Christian N. Kruse, Sören Schäfer, Felix Haase, Verena Mertens, Henning Schulte-Huxel, Bianca Lim, Byungsul Min, Thorsten Dullweber, Robby Peibst, Rolf Brendel

**Affiliations:** 1 Institute for Solid State Physics, Leibniz Universität Hannover, Appelstrasse 2, 30167 Hannover, Germany; 2 Institute for Solar Energy Research Hamelin (ISFH), Am Ohrberg 1, Emmerthal, 31860 Germany; 3 Institute Electronic Materials and Devices, Leibniz Universität Hannover, Schneiderberg 32, 30167 Hannover, Germany

**Keywords:** Energy science and technology, Renewable energy, Solar energy, Photovoltaics, Electronic devices

## Abstract

We present a simulation-based study for identifying promising cell structures, which integrate poly-Si on oxide junctions into industrial crystalline silicon solar cells. The simulations use best-case measured input parameters to determine efficiency potentials. We also discuss the main challenges of industrially processing these structures. We find that structures based on p-type wafers in which the phosphorus diffusion is replaced by an n-type poly-Si on oxide junction (POLO) in combination with the conventional screen-printed and fired Al contacts show a high efficiency potential. The efficiency gains in comparsion to the 23.7% efficiency simulated for the PERC reference case are 1.0% for the POLO BJ (back junction) structure and 1.8% for the POLO IBC (interdigitated back contact) structure. The POLO BJ and the POLO IBC cells can be processed with lean process flows, which are built on major steps of the PERC process such as the screen-printed Al contacts and the $\text{Al}_\text{2 }\text{O}_\text{3 }/\text{SiN }$ passivation. Cell concepts with contacts using poly-Si for both polarities ($\text{POLO}^2$-concepts) show an even higher efficiency gain potential of 1.3% for a $\text{POLO}^2$ BJ cell and 2.2% for a $\text{POLO}^2$ IBC cell in comparison to PERC. For these structures further research on poly-Si structuring and screen-printing on p-type poly-Si is necessary.

## Introduction

Passivating contacts enable selectivities with low recombination currents and contact resistances^1^ and therefore allow higher cell efficiencies than conventional diffused contacts, as can be found in passivated emitter and rear cells (PERC). One prominent passivating contact scheme, the heterojunction between amorphous (a-) H-rich Si and crystalline (c-) Si, provides the base for highly-efficient industrial double-side contacted cells with efficiencies up to 25.1%^2^ and is implemented in a lab-type interdigitated back contact (IBC) cell with the current Si world record efficiency of 26.7%^3^. Another prominent passivating contact scheme, which we denote as “Poly-silicon on oxide (POLO) junctions”, consists of a stack of an interfacial oxide and a doped (partially) crystalline Si layer. Its potential advantage as compared to a-Si:H/c-Si heterojunctions is that it is high-temperature stable and thus compatible with the manufacturing processes of today′s mainstream solar cell technology. Solar cell concepts featuring POLO or related junctions achieved high efficiencies up to 26.1% (26.0%) in lab-type IBC (double-side contacted)^3,4^ cells. In comparison, solar cell manufacturers are mainly producing mono-facial PERC and bifacial PERC+ solar cells^5^ with efficiencies around 22.5%^6^. However, although numerous manufacturers have started (pilot) production of solar cells featuring POLO junctions or are producing similar cell concepts on rather small scale since some years with impressive results^7–9^, these approaches are in strong competition with PERC and PERC+ as the “conventional” current mainstream. Some reasons for this hard standing are an increased process complexity and an uncertainty on the efficiency potential of the respective new cell structures. In this paper, we analyze different options for a lean POLO implementation into new and existing solar cell structures and compare these alternatives by means of a simulation-based study. Structures featuring POLO junctions have already been the subject of experimental and simulation-based studies^1,4,10–12^. However, due to the different experimental equipment, simulation approaches, input parameters and focus of the studies, the efficiency values extracted from these studies are not comparable. For some specific cell structures and specific realization routes, experimental building blocks have been evaluated, e.g. in Ref.^11^. Here, we focus on good comparability of the simulated efficiency between all analyzed cell structures and discuss the main processing challenges to give an impression of the expected process complexity. Besides the systematic screening of numerous cell structures, we also compare them in-depth based on their full Synergistic Efficiency Gain Analysis (SEGA). In addition, the simulation base is used to assess the sensitivity of the discussed solar cell structures to bulk lifetime variations. The goal of our study is to identify cell structures that show a high efficiency potential along with potentially lean process flows. From this discussion, a possible roadmap for industrializing p-type c-Si solar cells using POLO junctions is derived. (The experimental realization of the most promising cell structures will be the subject of future work.).

## Simulation method

Two important aspects when evaluating new cell structures for their potential economic benefit in large-scale production are the process complexity and the efficiency gain compared to proven concepts. To estimate the potential efficiency gain, we perform numerical device simulations using the Quokka^13^ implementation of the conductive boundary model^14^. These simulations require the knowledge of the resistive and recombination properties for the bulk, the passivated surfaces and the contacts as well as a charge-carrier generation profile for each structure. For a good comparability between all analyzed cell structures we choose equal input parameters for equal or comparable properties of the respective structures. For example, the recombination at a 200 nm thick n-type poly-Si junction is always assumed to be $J_\text{0 }= 3 \text{fA } / \text{cm}^2$ although in reality this $J_\text{0 }$ can vary for different cell structures due to the influence of other process steps on the passivation quality. In general, we choose best-case measured parameters achieved in lab-type production with industrial-type processes. We confine our analysis to screen-printed contacts, because most industrial solar cells are produced using screen-printing. Combining all these best-measured parameters into a hypothetical simulated cell gives an estimate for cell efficiencies to be expected in the next years for cells with POLO junctions and for PERC+ cells. All input parameters are detailed in Table 1.

**Table 1:** Electronic input parameters for the device simulations.

Parameter	Recombination	Resistive
p-type (B) bulk	$\tau _\text{n0 }$ = 2000 $\upmu \text{s }$	$\rho _\text{b }$ = 0.9 $\Omega \ \text{cm }$
	$\tau _\text{p0 }$ = 20,000 $\upmu \text{s }$	
n-type (P) bulk	$\tau _\text{b }$ = 22,000 $\upmu \text{s }$	$\rho _\text{b }$ = 3 $\Omega \ \text{cm }$
Pas. on p-Si (plan.)	$J_\text{0 }$ = 1$\text{fA } / \text{cm}^2$	n.a.
Pas. on p-Si (text.)	$J_\text{0 }$ = 3$\text{fA } / \text{cm}^2$	n.a.
Pas. on n-Si	$J_\text{0 }$ = 3 $\text{fA } / \text{cm}^2$	n.a.
emitter P-diff.	$J_\text{0 }$ = 22 $\text{fA } / \text{cm}^2$^25^	$R_\text{s }$ = 133 $\Omega /\square$^25^
selective P-diff.	$J_\text{0 }$ = 100 $\text{fA } / \text{cm}^2$	$R_\text{s }$ = 95 $\Omega /\square$
emitter B-diff.	$J_\text{0 }$ = 14 $\text{fA } / \text{cm}^2$^26^	$R_\text{s }$ = 135 $\Omega /\square$^26^
selective B-diff.	$J_\text{0 }$ = 110 $\text{fA } / \text{cm}^2$^27^	$R_\text{s }$ = 95 $\Omega /\square$^27^
n-type POLO	$J_\text{0 }$ = 3 $\text{fA } / \text{cm}^2$^22^	$R_\text{s }$ = 50 $\Omega /\square$
p-type POLO	$J_\text{0 }$ = 5 $\text{fA } / \text{cm}^2$	$R_\text{s }$ = 200 $\Omega /\square$
Ag on P cont.	$J_\text{0 }$ = 1400 $\text{fA } / \text{cm}^2$	$\rho _\text{c }$ = 1.5 $\mathrm {m}\Omega \ \text{cm}^2$
Al on Si cont.	$J_\text{0 }$ = 400 $\text{fA } / \text{cm}^2$^28^	$\rho _\text{c }$ = 1.3 $\mathrm {m}\Omega \ \text{cm}^2$
Ag on B cont.	$J_\text{0 }$ = 740 $\text{fA } / \text{cm}^2$^29^	$\rho _\text{c }$ = 2 $\mathrm {m}\Omega \ \text{cm}^2$^30^
Ag on n-POLO cont.	Same as n-POLO	$\rho _\text{c }$ = 0.9 $\mathrm {m}\Omega \ \text{cm}^2$
Ag on p-POLO cont.	Same as p-POLO^31^	$\rho _\text{c }$ = 5 $\mathrm {m}\Omega \ \text{cm}^2$

All parameters without a reference are ISFH internal measurements.

**Figure 1:**
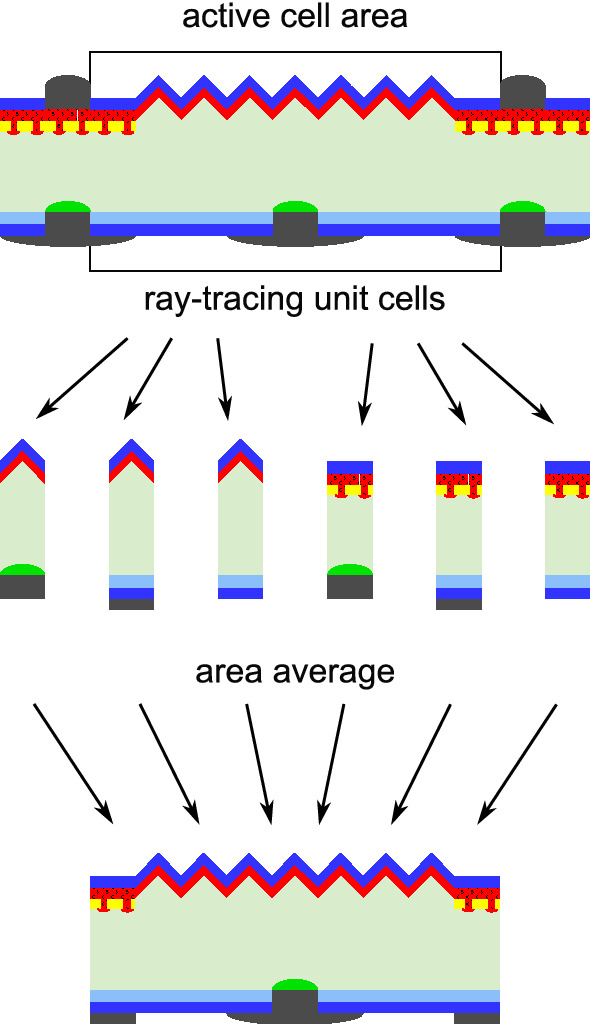
Ray-tracing approach used for the determination of generation profiles. From top to bottom: full cell structure with active cell area marked, the six unit cells required for the optical simulation of the example structure and area averaging for a charge carrier generation profile of the active cell area.

For the optical performance of each structure we employ ray-tracing simulations using the SUNRAYS ray-tracer^15^ with optical parameters chosen according to Refs.^16–18^. This program does, however, not allow simulating the complex unit cell shown at the top of Fig. 1. Therefore, we resolve the complex optical structure into simple unit-cells shown in the second row of Fig. 1 and simulate them separately. Each of these simulations yields a charge-carrier generation rate. Underneath the metal no optical generation is assumed. The generation profiles are averaged according to their area fractions. It should be noted that we assume an external mirror with ideal specular reflectance on the rear side in the simulations to represent a measuring chuck or white module back-sheet. The effect of this mirror on the simulated cell efficiencies is comparable for all structures because they all have a textured front and a planar rear side. Note that while the area-averaging approach accounts for differences in front surface metallization and parasitic absorption in the poly-Si, it lacks the impact of multiple internal reflections at, e.g. metal contacts. However, we estimate the additional parasitic absorption at the contacts to be below 10% of the total parasitic absorption within the cell. In addition, all structures have similar light trapping schemes and, thus, no structure is optically favored.

## Investigated solar cell structures

**Figure 2:**
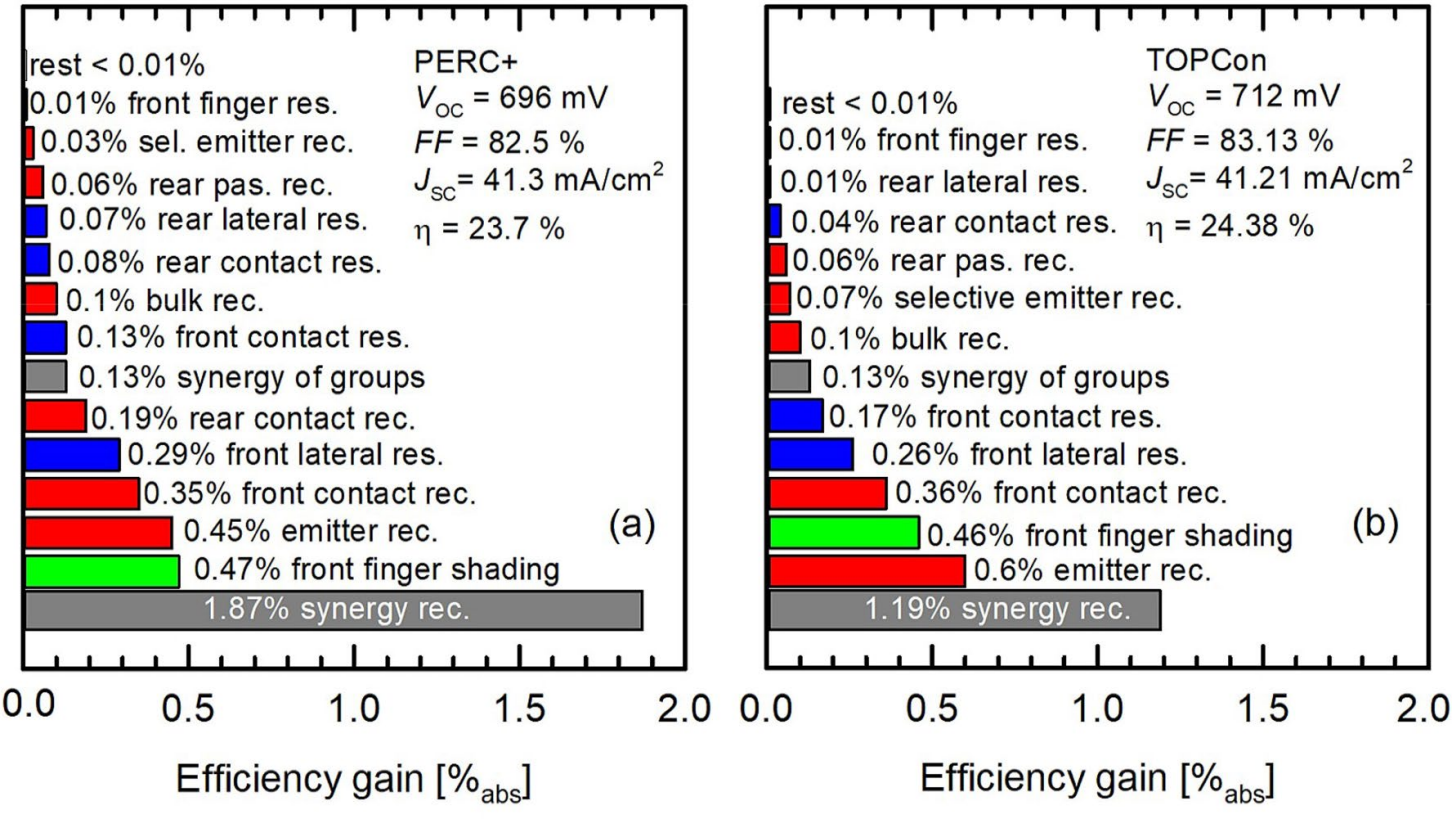
SEGA for (**a**) the reference PERC+ cell and (**b**) the TOPCon cell as a further benchmark for the alternative cell structures discussed in this study. Bars show potential efficiency gains due to complete suppression of different loss channels. Red bars are linked to recombination, green bars to optics, and blue bars to resistance. The gray bars show synergistic gains.

Most of the recently installed industrial solar cell production lines produce PERC and PERC+ solar cells, which have a high conversion efficiency and allow low production costs. However, the diffused contacts and front contact shading suggest an efficiency limitation at around 24%^19^. Figure 2a shows a synergistic efficiency gain analysis (SEGA)^20^ of an industrial-type PERC+ cell with best-measured input parameters given below. It should be noted that we only consider busbar-less cells in this work. The SEGA yields potential efficiency gains in solar cells by switching off the respective loss channel in the cell simulation and calculating the difference in conversion efficiencies. Synergistic effects between multiple loss channels are analyzed by switching off multiple loss channels simultaneously. The simulated efficiency is 23.7%, which agrees with the efficiency to be expected from PERC+ production lines within the next years given today’s efficiencies of around 22.5% and a 0.5% per year learning curve according to Ref.^21^. The efficiency gains due to a reduction of the front finger shading and a reduction of recombination in the emitter and at the front contact are the largest individual gains possible besides the synergistic gain between the extrinsic recombination channels. Therefore, the Ag contacts and the phosphorus diffusion are the bottlenecks on the road towards higher efficiencies. Here n-type POLO junctions offer better electrical performance. The so far most common approach for their implementation is the industrialization of the TOPCon^8,12^ cell structure. The corresponding SEGA is shown in Fig. 2b. For the input parameters assumed in this study, the resulting simulated efficiency is 24.4%, i.e., 0.7% higher than that for PERC+. This difference is mainly a consequence of the mitigation of the contact recombination at the electron-collecting contact, the lower $J_\text{0 }$of the B-doped front emitter, and the synergy between both loss channels. We acknowledge that our simulated efficiency for industrial TOPCon has already been surpassed experimentally: To our knowledge, the highest ISO 17025/IEC 6094 calibrated efficiency reported so far is 24.8%^9^. Obviously, it is possible to optimize single components further than assumed in Table 1. However, as discussed below, it still has to be shown that all possible measures for the optimization of a record cell (e.g. extremely deep emitters) are compatible with mass production. The median efficiency in production is currently around 23%^8^. It is still not yet clear whether TOPCon is the best suited cell structure for an integration of POLO junctions into industrial production.

**Figure 3:**
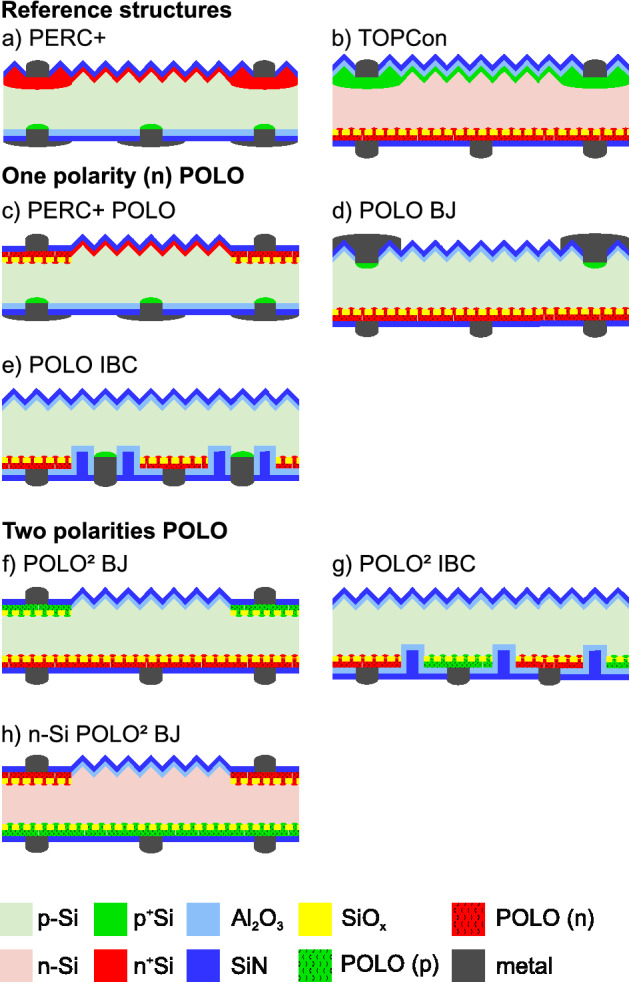
Cell structures discussed in this study. Top row: reference structures (**a**) the PERC+ cell and (**b**) the TOPCon structure. 2nd and 3rd row: concepts featuring n-type POLO junctions and Al contacts: (**c**) PERC+ POLO, (**d**) POLO BJ and (**e**) POLO IBC. 4th and 5th row: concepts featuring POLO junctions for both polarities (**f**) based on p-type Si in BJ configuration, (**g**) based on p-type Si in IBC configuration and (**h**) based on n-type Si in BJ configuration.

Therefore, we investigate six alternative structures featuring POLO junctions and compare their performance potential and processing complexity with that of PERC+ cells and with a TOPCon^8,12^ cell structure. It should be noted that these are cell structures we regard as interesting candidates for industrial integration after preliminary simulations and discussion, which are not shown here. There are, of course, many other possible cell structures and our list does not aim at being all-encompassing. In particular we focus our discussion on p-type doped Si solar cells, because it allows the re-utilization of established production steps. The six cell structures investigated are shown in Fig. 3 along with two reference structures. (a) the reference PERC+ cell, (b) the reference TOPCon cell based on n-type Si with a diffused boron emitter and a full-area n-type POLO junction on the rear, (c) the PERC+ POLO structure, which is a PERC+ cell with n-type POLO under the front contacts, (d) the POLO BJ structure, which features Al-alloyed (Al-p+) contacts on the front and a full-area n-type POLO on the rear side^10^, (e) the POLO IBC structure, which is an IBC concept featuring Al-p+ and n-type POLO junctions^10,22^, (f) the $\text{POLO}^2$ BJ structure, which features local p-type POLO junctions on the front side and a full-area n-type POLO emitter on the rear^11^, (g) the $\text{POLO}^2$ IBC structure, which is an IBC concept with POLO junctions for both polarities^4^ and (h) the n-Si $\text{POLO}^2$ BJ structure, which is the same structure as (f) but with reversed polarities. The six cell structures (c) through (f) feature either only one n-type poly-Si contact and an Al-p+ contact (POLO cell) or two POLO junctions ($\text{POLO}^2$ cells) in either front junction (FJ), back junction (BJ) or interdigitated back contact (IBC) configuration. We acknowledge the pioneering work of other groups (including companies) on many of these or related cell structures. For example, although not all details have been disclosed, the concept of TetraSun^23^ was reminiscent of the n-Si $\text{POLO}^2$BJ structure, and SunPower′s Maxeon technology^7,24^ seems to correspond to a n-Si $\text{POLO}^2$IBC structure.

It should be noted that for all structures either the structured layers underneath the contacts (selective emitter or POLO) are wider than the metal contact or the metal is wider than the LCO in case of Al contacts. This is due to alignment tolerances required for screen-printing, which we consider in our simulation study. The Ag contacts for which we assume a printing width of 30 $\upmu \text{m }$ are printed on 75 $\upmu \text{m }$-wide stripes of either POLO layers or the laser-doped selective emitter. For the Al contacts we assume an LCO width of 13 $\upmu \text{m }$ and 50 $\upmu \text{m }$-wide Al fingers. For all structures we individually optimize the front contact pitches. For the IBC cells we assume an emitter coverage of 80%. For the rear side, smaller contact pitches are in most cases preferable over larger pitches due to reduced resistance contributions. We, therefore, choose a rear contact pitch of 0.5 mm for all cells.

## Electronic input parameters

The electronic input parameters chosen in this work are values measured mostly on reference samples manufactured with lab-type processes that can be transferred to industrial-scale production. Where possible we choose values measured at ISFH because this assures the best consistency between the different parameters. All input parameters are shown in Table 1. Values without a reference are unpublished ISFH internal measurements. At ISFH we measure the lifetime of non-metallized samples using a WCT-120 lifetime tester by Sinton instruments^32^ and determine the surface recombination using the in-built Kane–Swanson^33^ analysis tool. The recombination at metallized surfaces is determined by comparing samples with different metallization fractions and fitting the respective lifetime, measured by photoluminescence emission, using Quokka. Bulk and sheet resistances are determined using a four-point probe setup whereas contact resistivities are determined using the transfer-length-method (TLM)^34,35^. It should be noted that some values differ from the ones used in our recently published paper in Ref.^11^, because they were either just recently measured or a publication exists for a similar value, which makes it easier for the reader to check the experimental details of the input parameters.

The boron-doped Czochralski-grown material that we use in our analysis was measured at ISFH with $\rho _\text{b }$ = 0.9 $\Omega \ \text{cm }$ and mid-gap Shockley–Read–Hall lifetime parameters $\tau _\text{n0 }$ = 2000 $\upmu \text{s }$ and $\tau _\text{p0 }$ = 20,000 $\upmu \text{s }$. The phosphorus-doped n-type material was also measured at ISFH with $\rho _\text{b }$ = 3 $\Omega \ \text{cm }$ and an injection-independent SRH lifetime of $\tau _\text{b }$ = 22,000 $\upmu \text{s }$. For the surfaces passivated with an $\text{Al}_\text{2 }\text{O}_\text{3 }/\text{SiN }$ stack we determine a $J_\text{0 }$ of 1 $\text{fA } / \text{cm}^2$ on planar and 3 $\text{fA } / \text{cm}^2$ for the passivation on textured p-type material. For n-type material we assume a passivation quality equal to that on p-type. For the phosphorus-diffused surfaces we determine $J_\text{0 }$ = 22 $\text{fA } / \text{cm}^2$ and $R_\text{s }$ = 133 $\Omega /\square$ for the inter-finger region^25^. For the selective doping underneath the front contacts we determine $J_\text{0 }$ = 100 $\text{fA } / \text{cm}^2$ and $R_\text{s }$ = 95 $\Omega /\square$. For the boron emitter in the TOPCon concept we take literature values for the shallow doped emitter^26^ with $J_\text{0 }$ = 14 $\text{fA } / \text{cm}^2$ and $R_\text{s }$ = 135 $\Omega /\square$ and the selective emitter^27^ with $J_\text{0 }$ = 110 $\text{fA } / \text{cm}^2$ and $R_\text{s }$ = 95 $\Omega /\square$. For the planar n- and p-type POLO junctions we assume 200 nm thickness for the calculation of the optical performance. We assume this thickness to be required for a screen-printed POLO contact to retain its recombination properties. Lower thickness values are possible in combination with other metallization schemes that are not considered here. For planar n-type POLO layers we measure a $J_\text{0 }$ of 3 $\text{fA } / \text{cm}^2$ and a sheet resistance of 50 $\Omega /\square$^22^. For p-type POLO layers we measure a $J_\text{0 }$ of 5 $\text{fA } / \text{cm}^2$ and a sheet resistance of 200 $\Omega /\square$. We determine the contact recombination of Ag contacts on a P-diffusion to $J_\text{0 }$ = 1400 $\text{fA } / \text{cm}^2$ in low injection. For the Ag on P contact we measure a contact resistivity of $\rho _\text{c }$ = 1.5 $\mathrm {m}\Omega \ \text{cm}^2$. For the Al-p+ contacts we determine $J_\text{0 }$ = 400 $\text{fA } / \text{cm}^2$ and $\rho _\text{c }$ = 1.3 $\mathrm {m}\Omega \ \text{cm}^2$. For the Ag contact on boron diffusion we again refer to the literature with $J_\text{0 }$ = 740 $\text{fA } / \text{cm}^2$^29^ and a $\rho _\text{c }$ = 2 $\mathrm {m}\Omega \ \text{cm}^2$^30^. For contacts on n-type POLO formed with firing-through pastes we observe that the contacts do not contribute to the recombination. These contacts were measured with a resistivity of $\rho _\text{c }$ = 0.9 $\mathrm {m}\Omega \ \text{cm}^2$. For p-type POLO the firing-through pastes yield high $J_\text{0 }$-values of around 250 $\text{fA } / \text{cm}^2$ as published by Mack et al.^36^. However, non-firing-through pastes on p-type POLO lead to insignificant recombination similar to n-type POLO contacts with a resistivity of $\rho _\text{c }$ = 5 $\mathrm {m}\Omega \ \text{cm}^2$ as measured at ISFH^4,31^.

## Results and discussion

Table 2 shows the simulated I–V parameters for each of the cell structures shown in Fig. 3. The results show clear trends in terms of increasing $V_\text{OC }$ and $J_\text{SC }$: $V_\text{OC }$ increases with the reduction of recombination losses at conventional contacts and diffusions. Consequently, the $\text{POLO}^2$ concepts show the highest $V_\text{OC }$ followed by the POLO concepts. However, POLO layers always lead to parasitic optical absorption. Therefore, cell structures featuring structured POLO at the front show lower $J_\text{SC }$ than those without POLO junctions. In addition, POLO layers on the rear also contribute to the parasitic absorption, leading to further $J_\text{SC }$ reduction for concepts featuring full-area rear side POLO layers. In the following discussion, we also comment on the bifaciality. For utility applications, it is mandatory to take this aspect into account when comparing different cell concepts on “equal footing”. Please note that the projection of the achievable bifaciality values for all cell structures is difficult and depends strongly on e.g. future paste development (Al lines widths, mitigation of spiking through thin poly-Si layers, ...). Thus, all assessments of this aspect here are based on today’s published values and our practical experienced-based projection of the potential for further improvement. For the discussion whether a cell concept is industrially viable, technological challenges, process complexity and cost of ownership aspects (e.g. tool and material costs, waste management aspects, ...) are also relevant. It is hardly possible to compare all of these aspects for all structures on equal footing. There is a large variety of processing techniques, e.g. for poly-Si deposition and structuring, with specific advantages and disadvantages. These process techniques can, in principle, be combined to numerous process sequences for each cell concept. Some of them are less mature than others, and some of them have not even been demonstrated experimentally. We therefore refrain here from proposing hypothetical (and partially speculative) sequences, which could be misleading for the assessment of the above-mentioned practical issues. Rather, we remark for all cell structures the major possible or proven technological challenges that we are aware of after  10 years of PERC and after  7 years of industrial POLO development at ISFH. For all structures with POLO junctions (regardless whether for one or two polarities), growth of the interfacial oxide, poly-Si deposition and a certain post-deposition high-temperature anneal are mandatory. For interfacial oxide growth, several methods like ozone oxidation^37,38^, wet chemical oxidation^12,38,39^, short thermal oxidation or the Plasma Enhanced Chemical Vapor Deposition (PE-CVD) of SiO_x_^40^ have been successfully demonstrated. The most viable option for industrial processing, e.g. with respect to throughput, reproducibility, homogeneity and robustness against contaminations, will have to be identified in pilot production. For the deposition of amorphous or polycrystalline Si, different methods like Low Pressure (LP-) CVD^38,39,41–43^, PE-CVD^12,44–48^, Atmospheric Pressure (AP-) CVD^49^, sputtering^50^ and e-beam evaporation^51^ have demonstrated excellent results. The process versatility of both, interfacial oxide growth and (poly-) Si deposition, hints to the robustness of the POLO concept. Apparently, the high temperature annealing reliably allows the system of crystalline Si, interfacial oxide and polycrystalline Si to re-configure into a low-energetic state. Regarding industrialization of the poly-Si deposition methods, the most recent ITRPV^52^ gives some indications, but we think it should be considered with some caution because new deposition techniques like sputtering are emerging and different cell concepts may favor different deposition techniques. Technological challenges are, besides aspects like homogeneity of Si layer thickness and (if present) in-situ dopant distribution, the absence of blistering and in particular the mitigation of wrap-around—also for nominal single-side deposition techniques like PE-CVD and AP-CVD. Alternatively, the process sequence can be designed in a way that it tolerates or even utilizes double-sided Si deposition^11^. For the post deposition high-temperature treatment, quartz tube furnace anneals (either in inert or oxidizing ambient or within a $\mathrm {POCl}_3$^44^ or $\mathrm {BBr}_3$ diffusion^45^) are currently standard. For some cell concepts, they might be replaced by so called “firing-only contacts”^49,53^ in the future, but the latter still have to demonstrate high selectivities (in particular low junction resistances), sufficiently low sheet resistances, compatibility with high-temperature screen-printing and so on.

**Table 2:** Simulation results.

Cell concept	$\eta$ [%]	$J_\text{SC }$ $\left[ \frac{\text{mA }}{\text{cm}^2}\right]$	$V_\text{OC }$ [mV]	$FF$ [%]
a) PERC	23.7	41.3	697	82.5
b) TOPCon	24.4	41.2	712	83.1
c) PERC+ POLO	24.1	40.9	712	82.8
d) POLO BJ	24.7	40.6	736	82.7
e) POLO IBC	25.5	41.7	733	83.4
f) $\text{POLO}^2$BJ	25.0	40.6	742	83.0
g) $\text{POLO}^2$IBC	25.9	41.7	742	83.5
h) n-Si $\text{POLO}^2$BJ	25.1	40.9	737	83.3

In the following, we discuss the different cell structures in detail: a) Our reference PERC+ cell shows a front-side efficiency of 23.7%. The SEGA is shown in Fig. 2. One should note that the bifaciality of PERC+ in the range of 80%^54^ allows for a significantly increased energy yield (10–20%^55^) for utility applications. This has to be taken into account when comparing the performance with other cell structures with higher or lower bifaciality.

From a technological point of view, a certain process sequence “standard” or “main route” has been established for PERC or PERC+^21^. It comprises at least one high temperature step ($\mathrm {POCl}_3$ diffusion for introduction of n+-type doping), while an ex-situ oxidation of the emitter is optional. Structuring for the selectively doped emitter is mainly performed via laser-doping from the phosphor silicate glass, which is an inexpensive process without the necessity of any waste management. A further technological advantage of the PERC+ structure is that Al alloying during firing forms the contact for the second polarity (holes) inexpensively. The rear side metallization furthermore only contains a limited amount of silver (for soldering pads, not for fingers). Thus, the PERC+ structure poses a tough benchmark (with respect to cost per watt, but also to Levelized Costs of Electricity) for any other cell concept.

b) A widely evaluated concept is the TOPCon concept based on an n-type Si wafer with n-type POLO on the rear and a diffused boron emitter on the front. We include this concept as a second reference. In our simulation study, it yields a front-side efficiency potential of 24.4%, i.e., 0.7% higher than that of the PERC+ cell. The bifaciality of industrial TOPCon cells is reported to be in the range of  80 %^56^, i.e., comparable to that of PERC+.

In terms of technology, the process sequence for the fabrication of a TOPCon cell strongly depends on the availability of real single-side Si deposition techniques (without wrap-around) and on the availability of firing-only contacts. Without the latter, two high temperature processes—one for $\mathrm {BBr}_3$ diffusion and one for POLO junction formation—are required. The reason is that the thermal budgets of both processes are typically quite different (unless “thick” interfacial oxide is used), and that the boron silicate glass and the boron doping from the rear has to be removed prior to the poly-Si deposition. Replacing the $\mathrm {BBr}_3$ diffusion by single-side boron doping techniques like AP-CVD deposition of boron-doped glasses could eliminate the latter necessity. The boron doping profile can be driven-in several micrometers in order to mitigate contact recombination on the front - in expense of process time. Selectively doped boron emitters are less mature than their phosphorus-doped counterparts, and most work in this direction is so far based on etch-back approaches^57^ rather than on laser doping. If the poly-Si deposition yields an intolerable wrap-around, the poly-Si has to be removed selectively from the already present boron front-side emitter. To enable this step, an alkaline-resistant etch-stop layer on the front side (deposited prior to poly-Si deposition) could be required. This layer could also serve as a diffusion barrier when doping the poly-Si on the rear in-situ via $\mathrm {POCl}_3$ diffusion^8^. A cost disadvantage of the TOPCon structure—as compared to the PERC+ cell—is that the Ag consumption on the rear is increased due to the necessity to print Ag fingers for contacting the poly-Si without alloying through it. Besides cost aspects, it is worth to mention that contacting of n+-type poly-Si with firing-through Ag pastes without degrading the passivation quality works fairly well. Since there are controversial statements on cost perspectives for n-type wafers from the industry, it is difficult from our perspective to assess this issue. Also the relevance of the often cited higher robustness of n-type material with respect to some metal contaminations^58^ is difficult to assess. Under “clean” process conditions, the bulk lifetime in p-type material (and, more important, the diffusion length) can be comparable to that of excellent n-type material (see our results above), but the conditions in industrial environment might be different^59^. Last but not least, the absence of Light Induced Degradation is currently an advantage of n-type material, which might vanish in the near future due to the availability of low [O$_\text {i}$] B-doped or of Ga-doped p-type material, as well as due to the readiness of industrial feasible permanent BO deactivation schemes^60^.

c) The PERC+ POLO cell replaces the selective emitter of the PERC+ cell with a local n-type POLO layer. This brings an efficiency gain of 0.3% due to a higher open-circuit voltage (+ 15 mV) and fill factor (+ 0.3%) because of the reduced recombination and contact resistance. This positive effect overcompensates the loss in the short-circuit current density ($-\,0.4\: \text{mA } / \text{cm}^2$) due to absorption and reflection by the planar POLO layer in the finger regions.

In terms of technology, the process sequence for the fabrication of a PERC+ POLO cell is identical to that of a PERC+ cell for passivation and all steps afterwards. However, the necessity to structure the poly-Si on the front can imply a significant increase in process complexity. The straightforward approach adds the steps interfacial oxide growth, full-area deposition of poly-Si, print of an etch mask, poly-Si removal in the inter-finger regions, removal of the etch mask + cleaning and, for organic barriers, implies the necessity of waste treatment. Laser processing, e.g. laser oxidation^11^, might avoid the latter aspect and even the necessity to deposit or to print an etch barrier. The most attractive option would be to deposit the poly-Si already structured, e.g. by using shadow masks^61^, laser transfer techniques or by utilizing local print of Si inks or liquid Si^62^. All of these approaches are non-standard processes, which need development before becoming an option for industrial production. The rather small simulated efficiency gain of 0.3% questions the overall advantage of this cell structure.

**Figure 4:**
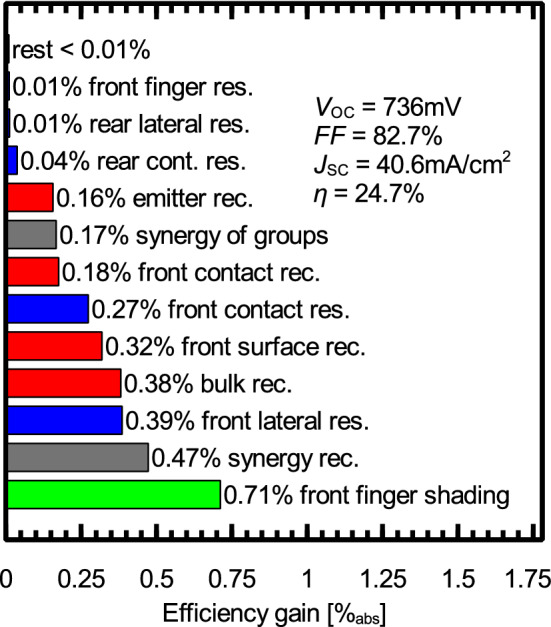
SEGA for the POLO BJ cell discussed in this study. Red bars show potential efficiency gains due to recombination, green bars gain due to optical losses and blue bars gain due to resistive losses. The gray bars show synergistic gains.

d) The POLO BJ cell yields an efficiency potential that is 1.0% above the reference PERC+ process. The SEGA of this structure is shown in Fig. 4. The larger shading losses of 0.7% (J_sc_: $-0.7\: \text{mA } / \text{cm}^2$) due to the 50 $\upmu \text{m }$ Al fingers are overcompensated by the gain in the open-circuit voltage (+ 39 mV) due to avoiding the recombination at the conventional P-diffusion and Ag contacts (0.45% and 0.35%, respectively). The disadvantage of larger shading will be reduced in the module, because a significant fraction of the light reflected from the finger will be redirected to the cell. Besides the shading loss, the resistance due to the large optimal finger spacing, and the synergies between the remaining recombination channels are the main bottlenecks of this structure. Regarding bifaciality, similar values as for TOPCon (i.e.,  80%) are expected and we measured values in this range, which will be published separately.

In terms of technology, this cell can possibly be manufactured with a lean process flow since no structuring of the poly-Si is required and the hole contact is formed by Al alloying during firing (as for the PERC+ cell). The latter aspect also implies comparable metallization costs for the POLO BJ cell as for the PERC+ benchmark. A small wrap-around of nominal single-sided poly-Si deposition techniques might be less detrimental than for e.g. the TOPCon structure since the poly-Si will not be in contact with highly doped regions of the opposite polarity. If in-situ doped firing-only contacts are available and the bulk lifetime is already sufficiently high even without any gettering step, a process sequence without any high temperature step in a quartz tube furnace might be possible. Achieving or maintaining a sufficiently high bulk lifetime, which is essential for all cell concepts and in particular for all back-junction concepts, is one of the technological challenges. To estimate the impact of different final lifetimes we run a variation of the bulk lifetime for all cell concepts based on p-type Si, which we show below. Printing narrow Al contacts (here 50 $\upmu \text{m }$ width) with good electrical properties and aligning them with respect to the laser contact openings in industrial production is another challenge, and requires further development. It is interesting to note that Al fingers as narrow as 50 $\upmu \text{m }$ were recently reported^63,64^. A further challenge is the reliable and, under industrial environment (automated handling, no cleanroom atmosphere), robust passivation of a lightly doped textured surface. Finally yet importantly, the inter-connection of the cells for module integration has to be developed. Besides geometrical constraints for possible soldering pads on the front due to shading, there are constraints on the SiN_x_ thickness in order to still fulfill antireflection purposes. Thus, metal spiking (i.e., by the Ag soldering pads) through the passivation layer cannot be mitigated by simply increasing the SiN_x_ thickness (as on the rear of a PERC+ cell). The comparably high efficiency potential of the POLO BJ structure comes with a potentially lean process flow and thus makes managing the above mentioned technological challenges a worthwhile research target.

**Figure 5:**
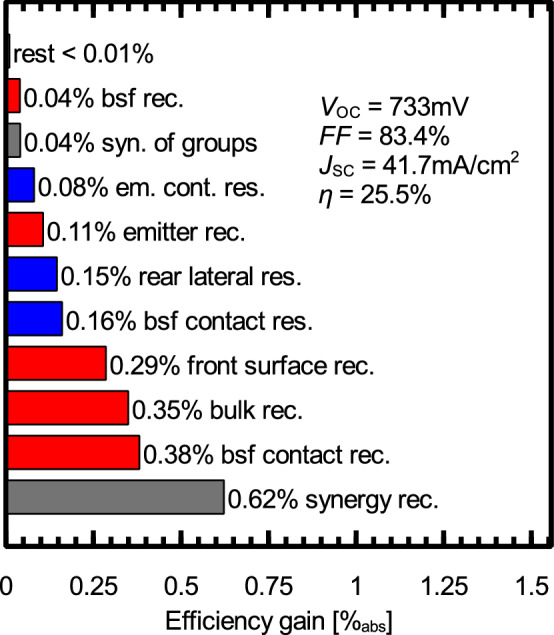
SEGA for the POLO IBC cell discussed in this study. Red bars show potential efficiency gains due to recombination, green bars gain due to optical losses and blue bars gain due to resistive losses. The gray bars show synergistic gains.

e) Conceptually, the POLO IBC has the same advantages as the BJ version discussed above, yielding a high gain in open-circuit voltage of + 36 mV compared to PERC+. The SEGA for this structure is shown in Fig. 5. When comparing the SEGAs of the POLO BJ and IBC structure, we see that the loss due to shading (0.71%) is completely avoided in the IBC design. In contrast the loss due to recombination at the Al-p+ contacts is increased because of the reduced contact spacing (+ 0.2%). The reduced contact spacing, however, also yields a reduction of the loss due to the lateral resistance between the contacts ($-\,0.24\%$). All effects combined yield an efficiency gain of 0.8% compared to the POLO BJ structure, which is 1.8% higher than for the reference PERC+ cell. The bifaciality of this cell structure can be expected to be slightly reduced from that of the abovementioned structures since both metal grids are on the rear. We expect a bifaciality factor of  70%.

In terms of technology, this cell structure also has the advantage of an inexpensive and elegant formation of the hole collecting contact by Al alloying. Unfortunately, it also combines challenges from the PERC+ POLO cell (necessity for structuring of the poly-Si) with challenges from the POLO BJ cell (requirement for high bulk lifetimes, passivation of lightly doped textured surfaces, avoidance of metal spiking through passivation layers, adapted cell interconnection scheme). Again, the increase of the SiN_x_ thickness on the rear is not a perfect measure for the mitigation of metal spiking (e.g. from the soldering pads) since the Ag paste still has to fire through the stack in the emitter (n+ POLO) regions. Assuming that these technological challenges can be solved, the high efficiency potential of this structure can also be combined with a lean process flow^22^.

**Figure 6:**
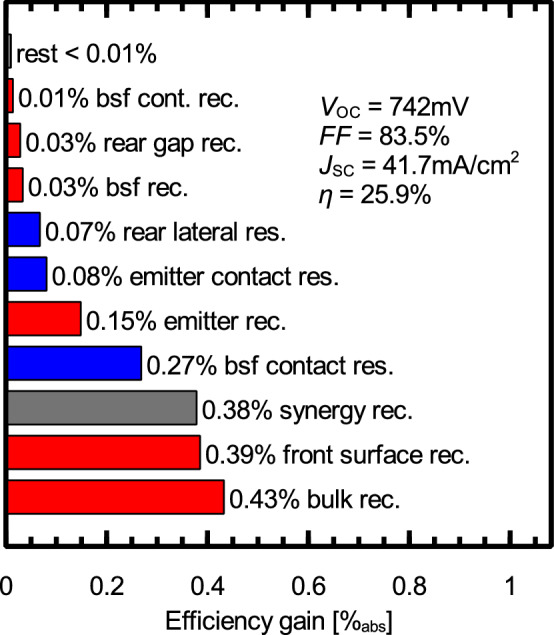
SEGA for the $\text{POLO}^2$ IBC cell discussed in this study. Red bars show potential efficiency gains due to recombination, green bars gain due to optical losses and blue bars gain due to resistive losses. The gray bars show synergistic gains.

f) and g) The POLO IBC concept is limited to 25.5% mainly by the screen-printed Al-p+ contacts. The logical next step is thus to also replace the Al-p+ contacts by p-type POLO junctions, promising an efficiency gain of 0.38%. We study both, f) the $\text{POLO}^2$ BJ and g) the $\text{POLO}^2$ IBC cell as improvements over the POLO BJ and IBC cells, respectively. Both cells are identical to the POLO BJ and IBC concepts except for the base contacts, which are replaced by p-type POLO junctions. The $\text{POLO}^2$ BJ structure shows an efficiency gain of 1.3% in comparison to PERC+ and 0.3% in comparison with the POLO BJ concept. In comparison to PERC+ this efficiency gain originates mainly from the large voltage gain of +45 mV and in comparison to POLO BJ from the reduced shading and recombination at the Al-p+ contacts. The IBC version of the $\text{POLO}^2$ concept yields the highest efficiency in our comparison with 25.9%, which is another 0.4% higher than the POLO IBC concept by avoiding the recombination at the Al-p+ contacts and 2.2% higher than PERC+. The SEGA for the $\text{POLO}^2$ IBC cell is shown in Fig. 6. We see that the main losses are due to recombination within the bulk and at the textured front surface. A further efficiency increase requires, thus, extensive research on Si material with reduced SRH defects and better surface passivation. All efficiency gains yield a final efficiency of 27.7%. The remaining margin to the efficiency limit of 29.6%^65^ is due to parasitic absorption, front surface reflection and imperfect light trapping, which do not show up in our analyses. Also the cell thickness and doping do not have the values required for the 29.6% limit. Bifaciality is expected to be lowest for $\text{POLO}^2$ IBC (60%), since the rear side of this structure features the metallization for both polarities as well as parasitically absorbing poly-Si on almost the entire area of the planar surface. Nevertheless, this cell structure will have the highest energy yield for any monofacial application such as rooftop, vehicle integrated PV and others.

In terms of technology, these $\text{POLO}^2$ structures add further challenges to those already mentioned for POLO IBC: the necessity to dope the poly-Si locally or at least single-sidedly, the necessity to contact p+-type poly-Si with high temperature screen-printing without alloying through it, and, for $\text{POLO}^2$ IBC, the necessity to electrically separate the n+-type and p+-type poly-Si regions^66–68^. For the first aspect, we proposed in Ref.^11^ the overcompensation concept, which can be applied for both $\text{POLO}^2$ BJ and $\text{POLO}^2$ IBC. Alternative approaches would be the single-sided or even structured deposition of doping sources (e.g. boron or phosphorus containing glasses^69^, doping inks^70^, masked ion implantation^68,71^) on initially intrinsic poly-Si, the single-sided or even structured deposition of in-situ doped poly-Si (structuring e.g. via shadow masks or inkjet printing of liquid silicon), or the extensive use of (structured) diffusion barriers. Many of the latter approaches are still in a very early stage of their development and have not proven their viability yet. For the second additional challenge (contacting of p+-type poly-Si via screen-printing), it will be essential to what extent the paste manufacturers can improve their products. We have reported our current status ($J_\text{0 }$ = 500 $\text{fA } / \text{cm}^2$, $\rho _\text{c }$= 5 $\mathrm {m}\Omega \ \text{cm}^2$) in Ref.^11^, and these results are consistent with those of other groups^36^. Since it could be a fundamental aspect that a certain amount of Al in the paste is required to form a low contact resistance to p+-type (poly-) Si^72^ while the Al always yields a certain degradation of the passivation quality, alternative metallization concepts like plating or Physical Vapor Deposition (PVD) might become more attractive for $\text{POLO}^2$ structures than they used to be for PERC+ cells. For the third additional challenge (separation of n+-type and p+-type poly-Si for $\text{POLO}^2$ IBC), different approaches can be used: oxidation of the poly-Si between the fingers^73^, introduction of a trench between the fingers^67,74–77^, or introduction of an initially intrinsic poly-Si region^4,66–68^. To summarize, both $\text{POLO}^2$ concepts are likely to require more time for industrial integration than the POLO BJ and IBC concepts. (h) The cell structures (c) through (g) discussed above use p-type Si wafers as does today’s mainstream PERC+ technology. Switching to n-type material brings a slight efficiency advantage due to the higher lifetime: The n-Si $\text{POLO}^2$ BJ concept shows an efficiency potential of 25.1%, 0.1% higher than in the p-Si concept, that is otherwise identical. The benefit of integrating n-type Si into cell concepts is small due to the slightly higher recombination at the p-type POLO junction (5 $\text{fA } / \text{cm}^2$ compared to 3 $\text{fA } / \text{cm}^2$ for n-type POLO).

In terms of technology, the challenges for n-Si $\text{POLO}^2$ BJ are—except for the fact that another passivation layer might be required for the lightly doped inter-finger regions on the front—comparable to those for the p-Si $\text{POLO}^2$ BJ cell.

## Sensitivity of the efficiency to bulk lifetime variations

**Figure 7:**
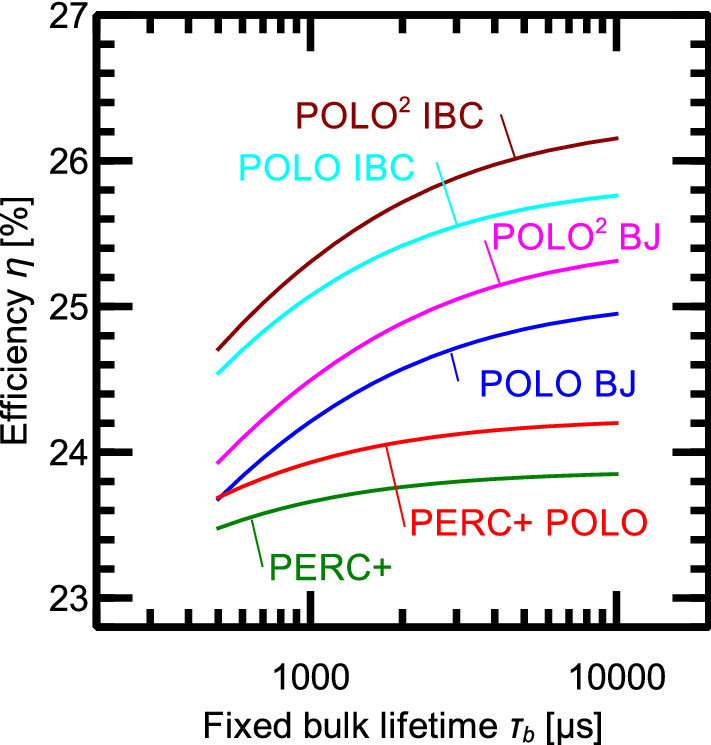
Conversion efficiency for the cell structures based on p-type Si discussed in this work as a function of an injection-independent bulk SRH lifetime.

The bulk lifetime is an important parameter for finding the best cell type with POLO junctions for industrial production. In general, the bulk lifetime depends on the cell process due to gettering effects and defect kinetics. This is especially true for p-type Si wafers. We, therefore, vary the bulk lifetime of the various cell types to analyze the sensitivity of the efficiency to bulk lifetime variations. The cell geometries are those optimized for high lifetimes. The optimized front finger pitch, however, depends mainly on the trade-off between resistance contributions and shading. Therefore, the variation of the optimum finger pitch with varying lifetime is small. Figure 7 shows the efficiencies of all cell types as a function of a fixed, i.e. injection-independent, SRH bulk lifetime. The lifetime has only a minor effect on the efficiencies of the PERC+ and PERC+ POLO concepts of less than 0.5% for lifetimes between 500 $\upmu \text{s }$ and 10,000 $\upmu \text{s }$. The benefit of integrating POLO into BJ or IBC p-type Si cells, however, depends strongly on the bulk lifetime. For a low lifetime of 500 $\upmu \text{s }$ the benefit of the POLO BJ and IBC are only 0.22% and 1.09%, respectively, and for the $\text{POLO}^2$ BJ and IBC concepts 0.48% and 1.26%, respectively. Compared to the expected efficiency gain at $\tau _\text{b }$ = 10,000 $\upmu \text{s }$, this is ca. 1.2% lower for the POLO concepts and 1.4% for the $\text{POLO}^2$ concepts and probably hardly justifies the increased process complexity. Thus, the economic feasibility relies on good quality Si and efficient gettering. In this context, it is interesting, that POLO layers also serve as gettering layers in high temperature steps^78^.

## Roadmap for future cell development

**Figure 8:**
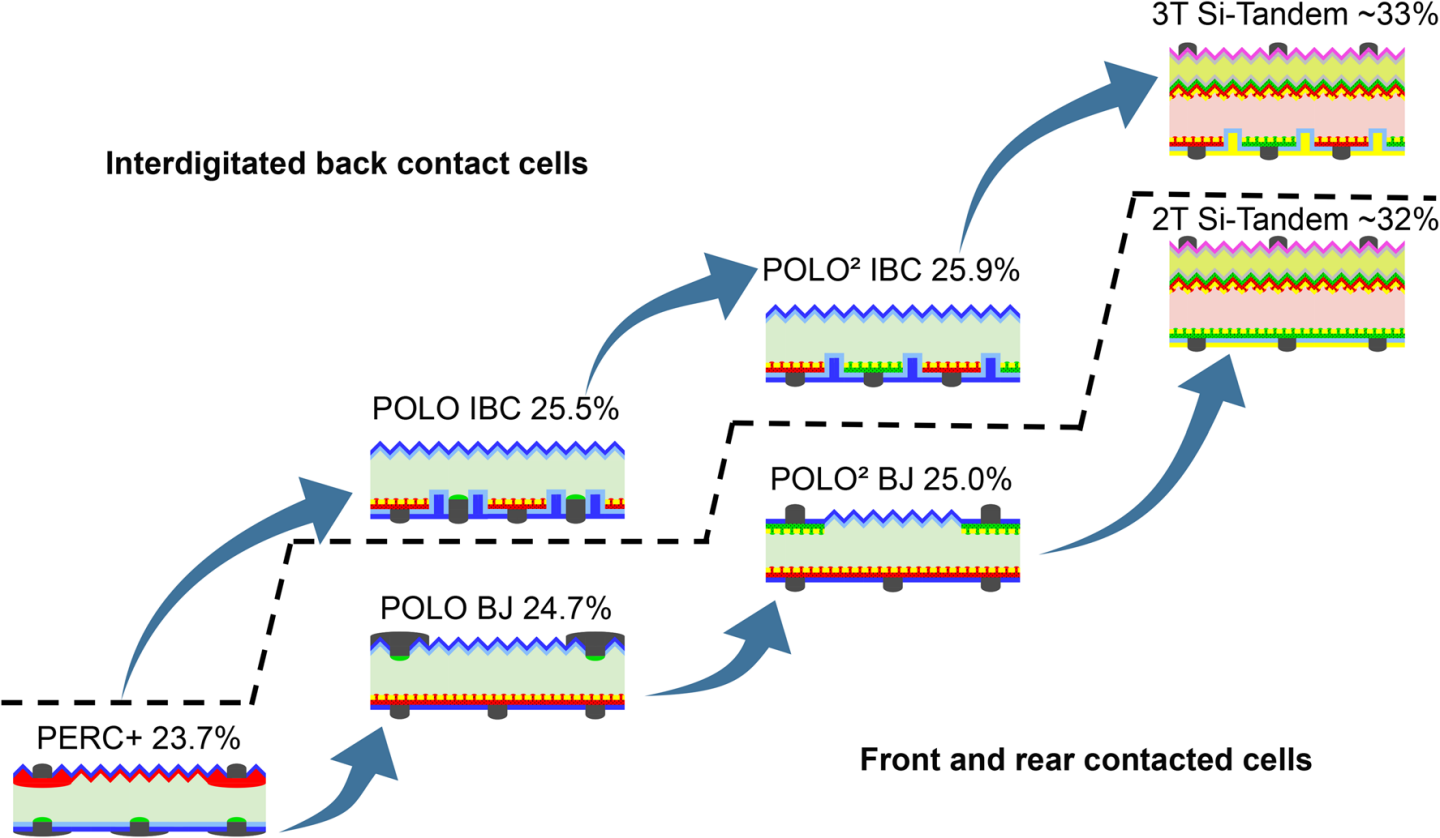
Roadmap for further cell development: Starting from our PERC+ cells we focus on the POLO IBC structure for industrial Si cells in the near future. We also exploit the large synergy between the POLO IBC and BJ concepts to also realize the BJ concept as it has the benefit of being compatible with conventional cell interconnection. As a further development step we work on the $\text{POLO}^2$ concepts, which require development in terms of POLO structuring and screen-printing on p-type poly-Si. The development step after the $\text{POLO}^2$ concepts is to combine the $\text{POLO}^2$ as bottom cells to perovskite tandem cells.

The results presented above can serve as a roadmap for a step by step improvement of crystalline Si solar cells using POLO junctions. Figure 8 shows this roadmap. The POLO BJ and IBC concept show a high efficiency gain of 1.0% and 1.8%, respectively, provided the high lifetimes measured on test structures can be transferred to the finished cell. Both concepts have potentially lean process flows with no and with one structuring process for the poly-Si, respectively. Potential process flows of both cells contain many process steps similar to that of the PERC+ process, which certainly eases the industrial integration. Therefore, starting from the PERC+ process, working towards the two POLO cells seems promising in terms of a fast integration of POLO junctions into industrial cells. Also there are large synergies between experimental developments of the POLO IBC and the POLO BJ cell. We actually started experimental work in this direction. For POLO BJ, we achieved an independently confirmed efficiency of 22.3 % within a (on our scale) very short development time of  1/2 year^79,80^. For POLO IBC, we achieved so far an open-circuit voltage of 711 mV and an efficiency of 22.6% on small area^81,82^. This $V_\text{OC }$ is still below the simulated value (due to a non-optimized Al-p+ contact), but already the highest experimental $V_\text{OC }$reported so far for p-type solar cells with Al-p+ contacts. We think that these first experimental achievements are promising and show that our simulation-based roadmap is reasonable. A promising next development target are industrial-type $\text{POLO}^2$ cells based on p-type Si both as IBC and BJ structure. We already fabricated a $\text{POLO}^2$ IBC cell with 26.1% efficiency^77^ using lab-type processes and evaporated contacts. The $\text{POLO}^2$ concepts are ultimately limited by their optical performance and material quality and of course the intrinsic transmission and thermalization losses. To address the latter loss channel, a further valuable research goal is the development of perovskite tandem cells with the Si $\text{POLO}^2$ concepts as bottom cells for 2- and 3-terminal devices. The analysis of these tandem cells is not part of this study but we estimate an efficiency of above 33% by adding an approximate 21% efficiency for the top and half of the efficiency simulated here for the bottom cell. The roadmap shown in Fig. 8 is one possibility for approaching highest efficiencies. It is, however, also possible to directly develop $\text{POLO}^2$ structures or implement tandem cells in earlier development stages like the POLO concepts or even at the current PERC+ state.

## Conclusion

In the simulation study presented here we analyzed various cell structures for their suitability for industrial integration. We simulated eight cell types: a PERC+ reference, a TOPCon reference, three cell concepts with n-type POLO and Al base contacts, and three cell concepts with POLO junctions for both polarities, two of which are based on p- and one based on n-type Si. The input parameters are chosen to represent measured best-case parameters achieved with processes relevant to industrial production. We simulated an efficiency gain compared to the reference PERC+ cell of 1.0% and 1.8% for the POLO BJ and IBC concepts, respectively, which feature n-type POLO emitters and Al base contacts. We further simulated an efficiency gain of 1.3% and 2.2% for the $\text{POLO}^2$ BJ and IBC concepts, respectively. We also analyzed the concepts based on p-type Si for their sensitivity to the bulk lifetime showing a 1.2% efficiency gain difference for the POLO concepts and 1.4% for the $\text{POLO}^2$ concepts for SRH bulk lifetimes ranging from 500 to 10,000 $\upmu \text{s }$. For the integration of POLO junctions into industrial production in the near future the POLO BJ and IBC concepts appear attractive. Both utilize the base contacts and passivation surfaces from PERC+ and can, therefore, build on process steps from existing production lines. The next development step is to replace the Al base contacts with p-type POLO junctions also in either BJ or IBC configuration. Even though the industrial integration of these concepts may be a few years down the road, the building blocks for their realization are currently under development^11,28^. Perovskite-Si tandem cells are still in a research state where their economic feasibility is not yet proven. Nevertheless, perovskite-Si tandem cells using the Si $\text{POLO}^2$ as bottom cells are an option for further improvement after the power losses in Si cells approach the intrinsic limit.

## References

[1] Hermle M, Feldmann F, Bivour M, Goldschmidt JC, Glunz SW (2020). Passivating contacts and tandem concepts: Approaches for the highest silicon-based solar cell efficiencies. Appl. Phys. Rev..

[2] Hanergy. Hanergy hits 25.11% efficiency with HJT cell. https://www.pv-magazine.com/2019/11/20/hanergy-sets-new-heterojunction-module-efficiency-record/ (2019).

[3] Green MA (2020). Solar cell efficiency tables (Version 55). Prog. Photovolt. Res. Appl..

[4] Haase F (2018). Laser contact openings for local poly-Si-metal contacts enabling 26.1%-efficient POLO-IBC solar cells. Sol. Energy Mater. Sol. Cells.

[5] Dullweber T (2016). PERC+: Industrial PERC solar cells with rear Al grid enabling bifaciality and reduced Al paste consumption. Prog. Photovolt. Res. Appl..

[6] International Technology Roadmap for Photovoltaics (ITRPV), PERC market share p. 43. https://itrpv.vdma.org/ (2019).

[7] Smith DD (2014). Toward the practical limits of silicon solar cells. IEEE J. Photovolt..

[8] Chen D (2020). 24.58% total area efficiency of screen-printed, large area industrial silicon solar cells with the tunnel oxide passivated contacts (i-TOPCon) design. Sol. Energy Mater. Sol. Cells.

[9] JinkoSolar. JinkoSolar has n-type mono cell verified at record 24.79% conversion efficiency. https://www.pv-tech.org/news/jinkosolar-has-n-type-mono-cell-verified-at-record-24.79-conversion-efficie (2020).

[10] Brendel, R. *et al.* Screening carrier selective contact combinations for novel crystalline Si cell structures. In *Proceedings of the 35th European Photovoltaic Solar Energy Conference and Exhibition*, 39–46. 10.4229/35thEUPVSEC20182018-1AO.2.6 (2018).

[11] Peibst R (2020). For none, one, or two polarities—how do POLO junctions fit best into industrial Si solar cells?. Prog. Photovolt. Res. Appl..

[12] Feldmann F, Bivour M, Reichel C, Hermle M, Glunz SW (2014). Passivated rear contacts for high-efficiency n-type Si solar cells providing high interface passivation quality and excellent transport characteristics. Sol. Energy Mater. Sol. Cells.

[13] Fell A (2013). A free and fast three-dimensional/two-dimensional solar cell simulator featuring conductive boundary and quasi-neutrality approximations. IEEE Trans. Electron Devices.

[14] Brendel R (2012). Modeling solar cells with the dopant-diffused layers treated as conductive boundaries. Prog. Photovolt. Res. Appl..

[15] Brendel R (1995). Coupling of light into mechanically textured silicon solar cells: A ray tracing study. Prog. Photovolt. Res. Appl..

[16] Reiter S (2016). Parasitic absorption in polycrystalline Si-layers for carrier-selective front junctions. Energy Proced..

[17] Schinke C (2015). Uncertainty analysis for the coefficient of band-to-band absorption of crystalline silicon. AIP Adv..

[18] Palik ED (1998). Handbook of Optical Constants of Solids.

[19] Min B (2017). A roadmap toward 24% efficient PERC solar cells in industrial mass production. IEEE J. Photovolt..

[20] Brendel R (2016). Breakdown of the efficiency gap to 29% based on experimental input data and modeling. Prog. Photovolt. Res. Appl..

[21] Dullweber T, Schmidt J (2016). Industrial silicon solar cells applying the passivated emitter and rear cell (PERC) concept—a review. IEEE J. Photovolt..

[22] Haase, F. *et al.* Transferring the record p-type Si POLO-IBC cell technology towards an industrial level. In *2019 IEEE 46th Photovoltaic Specialists Conference (PVSC)*, 2200–2206. 10.1109/PVSC40753.2019.8980960 (2019).

[23] Schultz-Wittmann, O. *et al.* High volume manufacturing of high efficiency crystalline silicon solar cells with shielded metal contacts. In *2016 32nd European Photovoltaic Solar Energy Conference and Exhibition*, 456–459. 10.4229/EUPVSEC20162016-2CO.4.5 (2016).

[24] Smith, D. D. *et al.* Generation III high efficiency lower cost technology: Transition to full scale manufacturing. In *2012 38th IEEE Photovoltaic Specialists Conference*, 001594–001597. 10.1109/PVSC.2012.6317899 (2012).

[25] Jäger, P., Baumann, U. & Dullweber, T. Impact of the thermal budget of the emitter formation on the pFF of PERC+ solar cells. In *AIP Conference Proceedings*, vol. 2147, 140005. 10.1063/1.5123892 (2019).

[26] Mihailetchi VD, Chu H, Lossen J, Kopecek R (2018). Surface passivation of boron-diffused junctions by a borosilicate glass and in situ grown silicon dioxide interface layer. IEEE J. Photovolt..

[27] Dahlinger, M., Eisele, S. J., Lill, P. C., Kohler, J. R. & Werner, J. H. Full area laser doped boron emitter silicon solar cells. In: *Conference record of the 38th IEEE Photovoltaic Specialists Conference*, 001029–001031. 10.1109/PVSC.2012.6317778 (IEEE, 2012).

[28] Dullweber T (2020). Evolutionary PERC+ solar cell efficiency projection towards 24% evaluating shadow-mask-deposited poly-Si fingers below the Ag front contact as next improvement step. Sol. Energy Mater. Sol. Cells.

[29] Lohmüller E, Lohmüller S, Wöhrle N, Belledin U, Wolf A (2018). BBr 3 diffusion with second deposition for laser-doped selective emitters from borosilicate glass. Sol. Energy Mater. Sol. Cells.

[30] Engelhardt, J. *et al.* Contact formation on boron doped silicon substrates from passivating PECV-deposited dielectric doping layers with anti-reflective properties by screen-printing Ag pastes for high-efficiency N-type silicon solar cells. In *Proceedings of the 31st European Photovoltaic Solar Energy Conference and Exhibition*, 351–354.10.4229/EUPVSEC20152015-2BO.4.4 (2015).

[31] Larionova, Y., Peibst, R. & Brendel, R. Screen print- and PVD based metallization schemes for POLO junctions. Metallization & Interconnection Workshop 2017, Konstanz, Germany (24.10.207).

[32] Sinton RA, Cuevas A (1996). Contactless determination of current–voltage characteristics and minority-carrier lifetimes in semiconductors from quasi-steady-state photoconductance data. Appl. Phys. Lett..

[33] Kane, D. E. & Swanson, R. M. Measurement of the emitter saturation current by a contactless photoconductivity decay method. In *Conference Record of the 18th IEEE Photovoltaic Specialists Conference*, 578 (Las Vegas, 1985).

[34] Berger, H. H. Contact resistance on diffused resistors. In *1969 IEEE International Solid-State Circuits Conference. Digest of Technical Papers*, vol. XII, 160–161. 10.1109/ISSCC.1969.1154702 (IEEE, 1969).

[35] Eidelloth S, Brendel R (2014). Analytical theory for extracting specific contact resistances of thick samples from the transmission line method. IEEE Electron Device Lett..

[36] Mack S (2017). Metallisation of boron-doped polysilicon layers by screen printed silver pastes. Phys. Status Solidi Rapid Res. Lett..

[37] Moldovan A (2015). Tunnel oxide passivated carrier-selective contacts based on ultra-thin SiO2 layers. Sol. Energy Mater. Sol. Cells.

[38] Peibst, R. *et al.* Implementation of n+ and p+ poly junctions on front and rear side of double-side contacted industrial silicon solar cells. In *Proceedings of the 32nd European Photovoltaic Solar Energy Conference and Exhibition*, 323–327. 10.4229/EUPVSEC20162016-2BO.3.2 (2016).

[39] Römer U (2014). Recombination behavior and contact resistance of n+ and p+ poly-crystalline Si/mono-crystalline Si junctions. Sol. Energy Mater. Sol. Cells.

[40] Huang Y (2020). Ultrathin silicon oxide prepared by in-line plasma-assisted N2O oxidation (PANO) and the application for n-type polysilicon passivated contact. Sol. Energy Mater. Sol. Cells.

[41] Gan, J. Y. & Swanson, R. M. Polysilicon emitters for silicon concentrator solar cells. In *Proceedings of 21st IEEE Photovoltaic Specialists Conference, 1990*, vol. 1, 245–250. 10.1109/PVSC.1990.111625 (1990).

[42] Stodolny MK (2016). n-Type polysilicon passivating contact for industrial bifacial n-type solar cells. Sol. Energy Mater. Sol. Cells.

[43] Duttagupta S (2018). MonoPoly cells: Large-area crystalline silicon solar cells with fire-through screen printed contact to doped polysilicon surfaces. Sol. Energy Mater. Sol. Cells.

[44] Yan D, Cuevas A, Bullock J, Wan Y, Samundsett C (2015). Phosphorus-diffused polysilicon contacts for solar cells. Sol. Energy Mater. Sol. Cells.

[45] Yan D, Cuevas A, Wan Y, Bullock J (2016). Passivating contacts for silicon solar cells based on boron-diffused recrystallized amorphous silicon and thin dielectric interlayers. Sol. Energy Mater. Sol. Cells.

[46] Tao Y (2016). Large area tunnel oxide passivated rear contact n -type Si solar cells with 21.2% efficiency. Prog. Photovolt. Res. Appl..

[47] Nogay G (2017). Interplay of annealing temperature and doping in hole selective rear contacts based on silicon-rich silicon-carbide thin films. Sol. Energy Mater. Sol. Cells.

[48] Stuckelberger J (2016). Passivating electron contact based on highly crystalline nanostructured silicon oxide layers for silicon solar cells. Sol. Energy Mater. Sol. Cells.

[49] Merkle, A. *et al.* Atmospheric pressure chemical vapor deposition of in-situ doped amorphous silicon layers for passivating contacts. In *35th European Photovoltaic Solar Energy Conference and Exhibition*, 785–791. 10.4229/35thEUPVSEC20182018-2DV.3.49 (WIP, 2018).

[50] Yan D, Cuevas A, Phang SP, Wan Y, Macdonald D (2018). 23% efficient p-type crystalline silicon solar cells with hole-selective passivating contacts based on physical vapor deposition of doped silicon films. Appl. Phys. Lett..

[51] Lossen, J. *et al.* Electron beam evaporation of silicon for poly-silicon/SiO2 passivated contacts. In *35th European Photovoltaic Solar Energy Conference and Exhibition*, 418–421. 10.4229/35THEUPVSEC20182018-2CO.10.5 (WIP, 2018).

[52] International Technology Roadmap for Photovoltaics. https://itrpv.vdma.org/ (2019).

[53] Ingenito A (2018). A passivating contact for silicon solar cells formed during a single firing thermal annealing. Nat. Energy.

[54] Dullweber T (2018). Present status and future perspectives of bifacial PERC+ solar cells and modules. Jpn. J. Appl. Phys..

[55] Stenzel, F. *et al.* Exceeding 23 % and mass production of p-Cz Q.ANTUM bifacial solar cells. In *36th European Photovoltaic Solar Energy Conference and Exhibition*, 96–99. 10.4229/EUPVSEC20192019-2BP.1.4 (WIP, 2019).

[56] Wu, W. *et al.* Development of industrial n-type bifacial topcon solar cells and modules. In *Proceedings of the 36th European Photovoltaic Solar Energy Conference and Exhibition*, 100–102. 10.4229/EUPVSEC20192019-2BP.1.5 (2019).

[57] Tao, Y. *et al.* Large-area n-type TOPCon cells with screen-printed contact on selective boron emitter formed by wet chemical etch-back. In *2017 IEEE 44th Photovoltaic Specialist Conference (PVSC)*, 1824–1827.10.1109/PVSC.2017.8366682 (IEEE, 2017).

[58] Schmidt, J. *et al.* n-type silicon – the better material choice for industrial high-efficiency solar cells? In *Proceedings of the 22nd EU-PVSEC*, 998–1001 (2007).

[59] Palinginis P (2019). Pioneering the industrialization of PERC technology: A review of the development of mono- and bifacial PERC solar cells at SolarWorld. Photovolt. Int..

[60] Walter DC (2014). Effect of rapid thermal annealing on recombination centres in boron-doped Czochralski-grown silicon. Appl. Phys. Lett..

[61] Tomasi A (2017). Simple processing of back-contacted silicon heterojunction solar cells using selective-area crystalline growth. Nat. Energy.

[62] Kiefer, F. *et al.* Inkjet printing as a new method for the preparation of POLO contacts. 2018 MRS Fall Meeting and Exhibit, Boston, MA, USA (2018).

[63] Tsuji, K. *et al.* Fine line Al printing on narrow point contact opening for front side metallization. In *AIP Conference Proceedings*, vol. 2147, 040019. 10.1063/1.5123846 (2019).

[64] Dullweber, T. *et al.* Bifacial PERC+ solar cells with conversion efficiencies above 22% and high energy yield. 12th International Photovoltaic Power Generation and Smart Energy Conference and Exhibition (SNEC2018), Shanghai, China (2018).

[65] Schäfer S, Brendel R (2018). Accurate calculation of the absorptance enhances efficiency limit of crystalline silicon solar cells with lambertian light trapping. IEEE J. Photovolt..

[66] Hollemann C (2020). Separating the two polarities of the POLO contacts of an 26.1%-efficient IBC solar cell. Sci. Rep..

[67] Reichel C (2017). Influence of the transition region between p- and n-type polycrystalline silicon passivating contacts on the performance of interdigitated back contact silicon solar cells. J. Appl. Phys..

[68] Young DL (2016). Interdigitated back passivated contact (IBPC) solar cells formed by ion implantation. IEEE J. Photovolt..

[69] Smith, D. D., Cudzinovic, M. J., McIntosh, K. R. & Mehta, B. G. Use of doped silicon dioxide in the fabrication of solar cells. https://patents.google.com/patent/US6998288B1/en (2004).

[70] Kiaee, Z. *et al.* Inkjet-printing of phosphorus and boron dopant sources for tunnel oxide passivating contacts. in *Proceedings of the 36th European Photovoltaic Solar Energy Conference and Exhibition, EU PVSEC 2019*, 187–191. 10.4229/EUPVSEC20192019-2BO.3.4 (2019).

[71] Römer U (2015). Ion implantation for poly-si passivated back-junction back-contacted solar cells. IEEE J. Photovolt..

[72] Kiefer F (2016). Structural investigation of printed Ag/Al contacts on silicon and numerical modeling of their contact recombination. IEEE J. Photovolt..

[73] Peibst, R. & Römer, U. Solar cell and a method for producing a solar cell with oxidised intermediate regions between polysilicon contacts. https://patents.google.com/patent/WO2016184840A2/en (2016).

[74] de Ceuster, D., Cousins, P. J. & Smith, D. D. Trench process and structure for backside contact solar cells with polysilicon doped regions. https://www.osti.gov/doepatents/biblio/1083920 (2013).

[75] Yang G, Ingenito A, Isabella O, Zeman M (2016). IBC c-Si solar cells based on ion-implanted poly-silicon passivating contacts. Sol. Energy Mater. Sol. Cells.

[76] Rienäcker M (2016). Recombination behavior of photolithography-free back junction back contact solar cells with carrier-selective polysilicon on oxide junctions for both polarities. Energy Proced..

[77] Haase F (2017). Interdigitated back contact solar cells with polycrystalline silicon on oxide passivating contacts for both polarities. Jpn. J. Appl. Phys..

[78] Krügener J (2017). Improvement of the SRH bulk lifetime upon formation of n-type POLO junctions for 25% efficient Si solar cells. Sol. Energy Mater. Sol. Cells.

[79] Min B (2020). A 22.3% Efficient p–Type Back Junction Solar Cell with an Al–Printed Front–Side Grid and a passivating n +—type polysilicon on oxide contact at the rear side. Solar RRL..

[80] Min, B. *et al.* POLO back junction: an elegant way to implement electron-collecting passivating contacts in p-Type industrial solar cells. In *2020 37th European Photovoltaic Solar Energy Conference and Exhibition*, 170–172. 10.4229/EUPVSEC20202020-2AO.6.4 (2020).

[81] Haase, F. *et al.* Fully screen-printed silicon solar cells with local Al-BSF base contact and a Voc of 711 mV. 37th European Photovoltaic Solar Energy Conference and Exhibition (07.09.2020).

[82] Haase, F. *et al.* Fully screen-printed silicon solar cells with local Al-BSF base contact and a Voc of 711 mV. *Progress in Photovoltaics: Research and Applications* under review (2020).

